# Integration of organics in nutrient management for rice-wheat system improves nitrogen use efficiency *via* favorable soil biological and electrochemical responses

**DOI:** 10.3389/fpls.2022.1075011

**Published:** 2023-01-05

**Authors:** Ajay Kumar Bhardwaj, Kapil Malik, Sukirtee Chejara, Deepika Rajwar, Bhaskar Narjary, Priyanka Chandra

**Affiliations:** Division of Soil and Crop Management, Central Soil Salinity Research Institute, Karnal, HR, India

**Keywords:** Nitrogen use efficiency, crop residue, legumes, green manure, soil carbon, redox potential, Sustainable nitrogen management index

## Abstract

**Introduction:**

The contrasting soil management in flooded-transplanted rice (*Oryza sativa*) and dry-tilled wheat (*Triticum aestivum*) poses a challenge for improving low nitrogen use efficiency (NUE) of the rice-wheat system. Integration of organics in nutrient management can bring in changes favoring efficient N uptake *via* changes in growing conditions and soil responses.

**Materials and methods:**

This study reported the results of a 15-year-long experiment on integrated nutrient management (INM) systems for rice-wheat cropping. The INM included substituting ~50% of chemical fertilizers *via* (i) including a legume crop (*Vigna radiata*) in the sequence and its biomass incorporation (LE), (ii) green manuring with *Sesbania aculeata* (GM), (iii) farmyard manure application (FYM), (iv) 1/3 wheat stubble *in situ* retention (WS), and (v) 1/3 rice stubble *in situ* retention.

**Results and Discussion:**

The INM strategies resulted in improved NUE compared to 100% chemical fertilizers (F). The INM had significantly higher net N mineralization and improved biological activity aligning with the NUE trends. The reductions in redox potential (Eh) and pH during rice season improved NUE under integrated management. Highly reduced conditions favored N mineralization and plant availability in form of ${\text{NH}}_{4}^{+}-\text{N}$ resulting in enhanced uptake efficiency, in rice crop. The soil organic carbon (C) significantly increased in INM, and an effect of the active C fractions was evident on the NUE of the wheat crop.

**Conclusion:**

The results showed that these INM strategies can immensely benefit the rice-wheat system *via* improvement in biological health along with electrochemical changes for flooded rice, and labile-C-assisted improvement in soil conditions for wheat.

## 1 Introduction

Rice (*Oryza sativa*) and wheat (*Triticum aestivum*) are two major crops of the world and play an essential role in the food and nutritional security of the ever-growing population of Asia, especially the south and southeast (Ladha et al., 2022). The rice-wheat cropping system is one of the biggest agricultural systems, practiced in an area nearing 13.5 million hectares (mha) in South Asia (Bhatt et al., 2016; Paudel et al., 2023). Indo-Gangetic Plain (IGP) which is also known as the Indian green revolution region has rice-wheat rotation followed in around 40% of its total area (Panigrahy et al., 2010). In its current form of management, with high nutrient use [>180 kg ha^-1^ nitrogen (N) use], high water use, and low resource use efficiency, the system fails sustainability criteria (Sandhu et al., 2016). The stagnating or declining yields of both crops have been attributed to the depletion of soil organic carbon (SOC) and soil fertility while no significant gain has been achieved in nutrient use efficiency. As there is little scope either for expanding the area under cultivation or using more natural resources, improving productivity and resource use efficiency is urgently needed. Continuous cultivation of both rice and wheat crops in the rotation has exhausted groundwater resources in irrigated areas, and aggravated pest problems. Both rice and wheat are exhaustive nutrient feeders, and the double cropping system has depleted the soil nutrient base (Banjara et al., 2022). Rice-wheat continuous cropping has also depleted the major nutrients (N, P, K, S) from the soil, created a nutrient imbalance, and led to deterioration in soil quality *via* SOC depletion (Bhardwaj et al., 2019; Debangshi and Ghosh, 2022). Long-term usage of selected chemical fertilizers without nutrient recycling declines soil fertility and productivity and introduces secondary and micronutrient scarcity (Dwivedi et al., 2016).

Nitrogen use efficiency is an important criterion for the assessment of a crop production system as it relates not only to productivity enhancement but also to environmental sustainability (Ding et al., 2018). The goal of NUE is to improve crop productivity *via* optimal nutrition for crops and reduce the N losses from the field (Dwivedi et al., 2016; Chen et al., 2018). To meet the poor production challenges in agriculture, intensive cropping practices were adopted, at the initiation of the green revolution but now, the declining response to added inputs has emerged as a key concern, especially for rice-wheat cropping systems (Bhardwaj et al., 2019). Rice and wheat have contrasting management with flooded-anaerobic soil management during transplanted rice season (July-October) and dry tilled-aerobic conditions during the wheat crop (November-April). Soil N conservation and efficient utilization is highly challenging under such conditions. Flooding of paddy (rice) soils quickly depletes soil oxygen and results in Ammonia NH_3_ volatilization while nitrate ( ${\text{NO}}_{3}^{-}-\text{N}$ ) leaching is a major loss during aerobic conditions (Farooq et al., 2022). An important strategy to enhance the efficiency of N is the integrated use of organic manures, and chemical fertilizers (Bhardwaj et al., 2020a; Bhardwaj et al., 2021). The basic concept underlying integrated nutrient management (INM) is the improvement of soil fertility while sustaining crop productivity and improving soil health through the judicious combination of chemical fertilizers, organic manures, and waste crop residues (Bhardwaj et al., 2019; Sharma et al., 2019). The common management practices under integrated nutrient management (INM) include the use of manures, compost, mulching, crop residues, diversified cropping systems, and cover crops (Vashisht et al., 2021).

**Figure 1:**
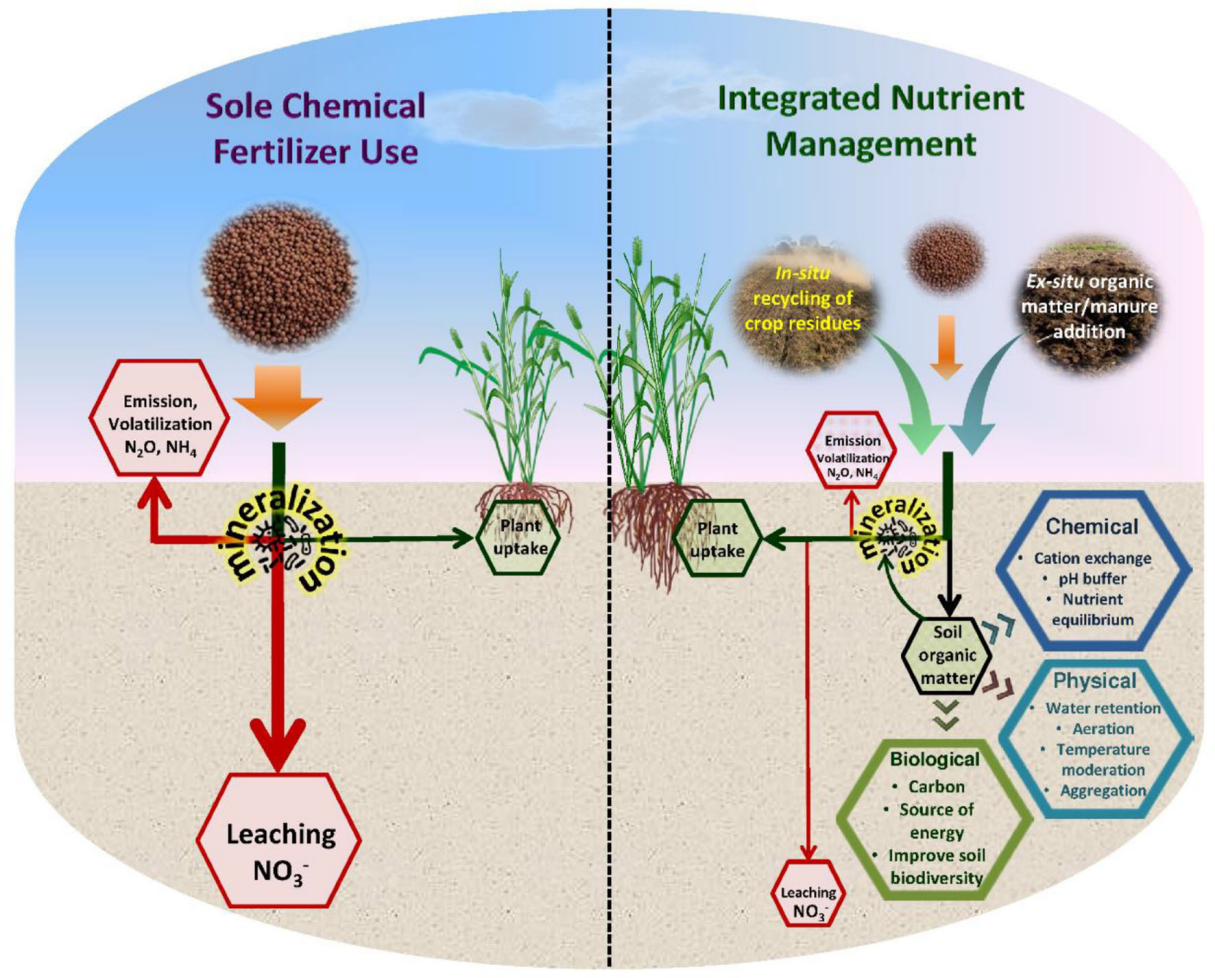
Contrasting influences of sole chemical fertilizer vs. integrated nutrient management on the nitrogen pools, mineralization, leaching, and volatilization fluxes.

Integrated nutrient management practice is recognized as a sustainable option for reinstating soil health, improving soil organic C, and sustaining the overall system productivity (**Figure 1**) (Dwivedi et al., 2016; Bhardwaj et al., 2019). It envisages replacing a part of chemical fertilizers with organic sources of nutrients, without adversely affecting yields. The possible organic options in the rice-wheat system include farmyard manure, green manure, legume cropping, cover crops, and the use of also crop residues of main crops which are often being burnt by farmers (Ladoni et al., 2015; Saha et al., 2018). India generates around 696.38 Mt (dry weight basis) of residue biomass annually, with 58.6% generated in the *kharif* or rainy season (June to October), 38.9% in the rabi or winter season (November to April/May), and 2.5% in the summer/dry season. Cereal crops generate about 364.27 million tonnes per year of crop residue. The gross crop residue produced from the rice and wheat crops per year was 156.8 million tonnes and 149 million tonnes, respectively (Venkatramanan et al., 2021; Kaur et al., 2022). With a moderate estimate of 0.4% N in the rice and wheat straw, there is a potential to recycle around 627 and 596 million kgs of nitrogen, respectively, through integration of rice and wheat residue in nutrient management. Cultivation of a legume mainly green gram (*Vigna radiata*) as a third crop during the summer season in the rice-wheat sequence has proved better in enhancing the system performance, and this can further help in saving chemical nutrient use (Bhardwaj et al., 2019). Green gram has occupied 34.00 lakh ha area and contributes 23.70 lakh tonnes in pulse production in the country (Naragund et al., 2022). Integrations of these crop residues into the nutrient management of rice-wheat, to recycle nutrients and reduce commercial fertilizer use, helps mitigate the environmental pollution issues due to burning of residue and excessive losses of nutrients from chemical fertilizers.

The gradual release of nutrients is a key feature of organic nutrient sources that distinguishes them from inorganic fertilizers. The slow-release characteristics make organic nutrient sources safer for the environment. However sole use of organic sources may not suffice to meet plant requirements, especially at critical stages when the plant nutrient demand is high (Dikgwatlhe et al., 2014; Xinqiang et al., 2014; Zhai et al., 2022). This is an important reason, besides labor-intensive operations, for farmers to prefer inorganic fertilizers compared to organic sources. Considering these limitations, a combination of organic alternatives and chemical fertilizers has been endorsed as a better option for crop production (Liu et al., 2009; Bhardwaj et al., 2019; Bhardwaj et al., 2020a; Bhardwaj et al., 2021). The continuous use of organic materials like crop residues, green manures, and farmyard manures decisively affects N dynamics in the soil-plant system (Singh et al., 2014). Legume crops in rotation not only fix biological N but also improve its availability to plants (Bhardwaj et al., 2020a; Bhardwaj et al., 2021), improve soil structure (Bhardwaj et al., 2019), and provide other minor and micronutrients which are otherwise skipped by farmers. The inclusion of organic sources in the rice-wheat system supplies carbon, a vital element for soil biological health. Farmyard manures provide macro as well as micronutrients and substantially improve soil quality (Chauhan et al., 2012). The rice-wheat system generates a large amount of residue which can be an important resource for recycling nutrients and adding C to the soil.

Strategies that integrate organics in nutrient management have been noted to sequester more C in soil (Bhardwaj et al., 2019), and have better N mineralization and plant availability (Bhardwaj et al., 2020a) than sole chemical fertilizer use. Though sufficient mineralization of N has been noted under INM for rice-wheat (Bhardwaj et al., 2020a; Bhardwaj et al., 2021), there is not much evidence of whether any gain in N use efficiency (NUE) is achieved or not. Often organic and conventional systems have shown similar NUE (Musyoka et al., 2017). In a 35-year study, by Hu et al. (2019), for rice-wheat system, the crop yields, as well as N-uptake, were higher in the combined application of mineral fertilizer and manures, but NUE was lower than manure alone. The partial factor productivity (PFP) of N has remained below the threshold in many Asian countries compared to developed ones (Heffer et al., 2017). Any level of gain in NUE would provide a significant advantage, economically and environmentally. We hypothesized that the changes in soil carbon fractions and other properties of soils with integration of organics might enhance the nutrient use efficiency *via* optimized moisture availability in the soil and nutrient uptake by the plants. While the C:N ratios of the organic amendments may play role in determining the NUE, the balance of improvements in soil properties (redox potential, pH) and the N release may provide advantage in improving NUE even with reduced fertilizer amounts. Therefore, in a 15-year-long field experiment on rice-wheat at the Central Soil Salinity Research Institute, Karnal, India, the present study was undertaken to evaluate five major INM strategies for rice-wheat cropping regarding NUE and to delineate the key factors leading to these responses.

## 2 Materials and methods

### 2.1 Experimental site

In 2005, a research project on integrated nutrient management (INM) in the rice-wheat cropping system was initiated at ICAR-Central Soil Salinity Research Institute (CSSRI), Karnal, India, which is located at 29.43° N latitude and 76.58° E longitude. The texture of the soil in the experimental field was sandy loam. The soil (0-15 cm) had a pH of 8.7, a bulk density of 1.43 Mg m^-3^, a cation exchange capacity of 9.5, and organic carbon of 3.2 g kg^-1^ at the start of the experiment. Semi-arid sub-tropical climatic conditions existed at the experimental location, with very hot summers, cool winters, and an average annual rainfall of 750 mm.

### 2.2 Experimental layout and treatments details

The treatments comprised a combination of different organic and inorganic inputs to provide for the nutrient requirements of rice (*Oryza sativa*)-wheat (*Triticum aestivum*) cropping system. The treatments were implemented in 5m × 4 m plots with four replications in completely randomized blocks. Five integrated nutrient management regimens were used in the study: LE- the residue of legume (*Vigna radiata*) taken as an opportunity crop during the lean period between wheat harvest in April and rice transplanting in July, GM-green manuring with *Sesbania aculeata*, FYM- farmyard manure application, WS- *in situ* wheat stubble retention and incorporation, and RS- *in situ* rice stubble retention and incorporation, along with reduced amount (~50% of recommended) of inorganic fertilizers. The following was the management schedule for these treatments:

- **O**: Rice followed by wheat was grown without any inorganic fertilizers or organic inputs.
- **F**: Rice (July-October) followed by wheat (November-April) was grown with 100% inorganic fertilizer input. The fertilizer application was done in three splits at time= 0, 21, and 42 days after transplanting. No organic inputs were given.
- **LE**: *Vigna radiata*, a leguminous crop, was planted as an “opportunity crop” between wheat and rice during the summer lean time. After the wheat harvest, the Vigna seeds were planted in the first week of April. After 60 days of seeding, pods were harvested in the first week of July, and the remaining plant biomass was mixed into the soil with a power tiller soon before rice transplanting. Rice was transplanted in July, while wheat was seeded in November, with fertilizer inputs cut by half (~50 percent).
- **GM**: During the lean season between wheat and rice, a green manure crop, *Sesbania aculeata*, was planted. Every year, after the wheat harvest, the green manure crop was sowed on or around May 20th. The green manure crop biomass was incorporated into the soil with a power tiller 35-40 days after sowing, right before rice transplantation. Rice was transplanted in July, while wheat was seeded in November, with fertilizer inputs cut by half (~50 percent).
- **FYM**: Farmyard manure (FYM) at the rate of 10 t ha^-1^ was incorporated in the soil just before soil puddling and transplanting of rice. Rice was transplanted in July and wheat was sown in November with reduced (~50%) fertilizer inputs.
- **WS**: At the time of wheat harvest, 30 cm of standing stubble (or 1/3 of the total straw) was retained. Before soil puddling in the first week of July, the stubble was dry plowed into the soil. Rice was transplanted in July, while wheat was seeded in November, with fertilizer inputs cut by half (~50 percent).
- **RS**: At the time of rice harvest, 30 cm of standing stubble (or 1/3 of the total straw) was kept. When wheat was sown in the second week of November, the stubble was dry-plowed into the soil. Rice was transplanted in July, while wheat was seeded in November, with fertilizer inputs cut by half (~50 percent).

The annual agricultural system consisted of rice (*Oryza sativa* L.) in the summer (July-October) and wheat (*Triticum aestivum* L.) in the winter (November-April). Dry plowing was done in the last week of June in all of the treatments, followed by puddling under water inundated conditions, to prepare the field for rice. 30-day-old nursery-raised rice seedlings (var. Pusa 44) were transplanted at recommended row spacing (20 cm) in the plots in the first week of July. Fields were flooded for pudding, and for the first month after transplanting, a minimum of 10 cm of standing water was maintained on the soil surface. During the wheat season, soils were dry-tilled with a power tiller before being row-sown with wheat-HD2967 seeds in the second week of November at a recommended row spacing of 15 cm. At roughly 1-month intervals, 3- 4 surface irrigations were applied. The soils were only saturated, with no standing water on the surface, for the second month, and the third month, irrigations were applied every week and the soils were only saturated, with no standing water on the surface. Irrigation was turned off two weeks before harvest. For both crops, inorganic fertilizer rates of 180, 26, and 42 Kg ha^-1^ were recommended for N, P, and K, respectively. Nitrogen were applied through urea in 3 equal split doses at t=0, 21, and 42 days after transplanting (rice)/sowing (wheat) whereas, full doses of phosphorus and potassium were applied through diammonium phosphate (DAP) and muriate of potash (MOP), respectively, at the time of transplanting (rice)/sowing (wheat). Furthermore, at the time of transplanting/sowing, ZnSO_4_ was applied as a micronutrient fertilizer at the required amount of 7 kg ha^-1^ exclusively in the F treatment. Every year, rice was harvested in the last week of October, and wheat in the last week of March.

### 2.3 Soil sampling and chemical analysis

Representative soil samples (0-0.15m depth) were collected yearly from the experimental field after wheat harvesting in mid-April. For each of the four replicates, two samples were drawn at random spots within a plot and then mixed. Composite samples (4 samples for each treatment) were air-dried in shade at room temperature, ground with the help of a pestle and mortar, and sieved through a 2 mm stainless steel sieve, for further analysis. The redox potential (Eh) of soils under each treatment was measured using a portable ORP (oxidation-reduction potential) meter (Lovibond, Amesbury, United Kingdom; model SD60) with 2mV accuracy, 0.1mV resolution, and redox measurement range of -1800 to1800 mV. For taking the Eh readings, the ORP meter was directly inserted into the treated soil to a depth of 6 cm and kept for 10 minutes to stabilize. The Eh was measured at 10-15 day intervals throughout the rice season. Simultaneously, the soil samples were collected using a stainless steel core sampler (0.05m × 0.05m). The samples were dried and the soil pH and EC (electrical conductivity) were determined in a 1:2 (soil: water) suspension with a digital multimeter (Eutech Instruments, Singapore; model PC510).

### 2.4 Soil organic carbon and its fractions

Bhardwaj et al. (2019) described a modified Walkley and Black’s quick titration method for determining oxidizable soil organic carbon. Briefly, in this approach, a 250 mL conical flask was filled with a 2 g sieved (0.5 mm) soil sample passed through a 0.5 mm sieve. The conical flasks were filled with 10 mL of 1N K_2_Cr_2_O_7_ and 20 mL of concentrated H_2_SO_4_. 100 mL distilled water, 10 drops of diphenylamine indicator, and 0.5 g NaF were added to the mixture. N/2 ferrous ammonium sulfate was used to back-titrate excess 1N K_2_Cr_2_O_7_. Simultaneously, a blank was run, and SOC was determined. Using 12N, 18N, and 24N H_2_SO_4_ (acid/aqueous ratios of 0.5:1, 1:1, and 2:1, respectively), different fractions of SOC were determined, including very labile (VLc), labile (Lc), less labile (LLc), and non-labile (NLc; recalcitrant). The VLc fraction was determined by reacting with 12N H_2_SO_4_; Lc carbon was determined by calculating the difference in oxidizable C determined with 18 N and 12 N H_2_SO_4_ (18 N H_2_SO_4_ minus 12 N H_2_SO_4_); the LLc fraction was determined by calculating the difference in oxidizable organic C extracted with 24N and 18N H_2_SO_4_ (24 N H_2_SO_4_ minus 18 N H_2_SO_4_. The difference between total oxidizable carbon and oxidizable carbon obtained with 24N H_2_SO_4_ was used to calculate the NLc proportion. The VLc and Lc forms constitute an active carbon pool, whereas LLc and NLc forms constitute a passive carbon pool.

### 2.5 Carbon management index

The carbon management index (CMI) is a model that demonstrates how specific land use impacts soil quality in comparison to control soil. The following formulae (Blair et al., 1995; Zhang et al., 2022a) were used to determine the index:


(i) CMI=CPI × LI × 100

Where CPI is the carbon pool index and LI is the lability index of the soil


(ii) $$\text{CPI}=\frac{\text{Total soil carbon in the treatment}\;\left({\text{g kg}}^{-1}\right)\;}{\text{Total soil carbon in the reference }\left({\text{g kg}}^{-1}\right)}$$

(iii) $$\text{LI}=\frac{\text{L in the treatment}}{\text{L in the reference}}$$

Where, L is the carbon lability of the soil, and was calculated as:


(iv) $$\text{L}=\frac{\text{Content of labile C}}{\;\text{Content of non labile C}}$$

### 2.6 Nitrogen mineralization

Ion exchange resin (IER) membranes were used to measure nitrogen availability (net mineralization) in soils during the growing season. Membrane strips of 2.5cm by 10cm were cut out of big commercially available sheets (cation and anion separately) (General Electricals, Watertown, MA, USA). The strips were charged by dipping and stirring for 1.2 hours in 0.5 molL^-1^HCl and then for 5 hours in 0.5 molL^-1^ NaHCO_3_. Finally, deionized water was used to rinse them. The resin strips were inserted into vertical slots cut into the treated soils and securely closed, ensuring that the strips made contact with the soil. Both cation and anion strips were planted 5 cm apart, left in the soil for 15 days, and then replaced with new strips immediately after removing the old ones. This procedure was followed throughout the cropping season. The strips were rinsed with deionized water after being removed from the soil to eliminate any remaining soil. Both cation and anion strips were preserved and carried in a vial for extraction of ${\text{NH}}_{4}^{+}-\text{N}$ and ${\text{NO}}_{3}^{-}-\text{N}$ in the laboratory. For extraction, 70 mL of KCl (2 mol L^-1^) was added to the vial with strips, which were agitated for 1 hour before being decanted into a scintillation vial. The extracts were tested for ${\text{NH}}_{4}^{+}-\text{N}$ and ${\text{NO}}_{3}^{-}-\text{N}$ using the Kjeltec 2200 (Foss, Hillerod, Denmark).

### 2.7 Enzymatic activity

The soil samples for enzymatic activity were drawn at the harvest of rice and wheat crops. The field-moist soil samples were immediately passed through a 2 mm sieve. Sub-samples were drawn for moisture determination. The moisture content of soil subsamples was determined by loss in weight after drying at 105°C for 48 h. The enzymatic activity was assayed for all treatment samples in duplicate and one in control. The enzymes urease, dehydrogenase, β-glucosidase, Acid phosphatase, and alkaline phosphatase were quantified according to the colorimetric analysis of the reaction products after sample incubation with the adequate substrate under standard conditions. Urease activity was performed by the method of Kandeler and Gerber, 1988; Hu et al., 2023, where urea was used as a substrate. The dehydrogenase activity was estimated according to the method of Casida et al., 1964; Ghosh et al., 2022, and determination was based on the reduction of triphenyl tetrazolium chloride (TTC). The β-Glucosidase activity was determined by the method of Eivazi and Tabatabai, 1988; Nakayama et al., 2023, and ρnitrophenyl-β-D-glucoside was used as a substrate. The absorbance of the products was measured using a spectrophotometer (Specord 200 plus, Analytic Jena, Germany). Acid and alkaline phosphatase activity of the soil was evaluated by the method of Tabatabai and Bremner, 1969; Boteva et al., 2022. Toluene solution modified universal buffer, 0.5 ml CaCl_2_was added in soil and swirled. The developed yellow color intensity was measured at 440 nm wavelength. The Geometrical Mean (GM_enz_) of enzyme activity integrates information from all enzymes calculated for each treatment using the following equation;


(v) $${\text{GM}}_{\text{enz}}={\left(\text{Urease }\times \text{ dehydrogenase }\times \text{ β}-\text{glucosidase }\times \text{ Acid phosphatase }\times \text{ alkaline phosphatase}\right)}^{1/5}$$

### 2.8 Culturable microbial population and diversity

The serial dilution method was used for the determination of culturable microbial count in the rhizosphere soils and was expressed in colony-forming units (CFUs) per gram soil. The collected rhizospheric soils were serially diluted by suspending 10g of soil in 90 ml sterilized distilled water followed by successive resuspension up to 10^-8^. For bacterial, fungal and actinobacterial counts nutrient agar (Peptone 5.0; Yeast Extract 2.0; Sodium Chloride 5.0; Agar 15.0), Potato Dextrose Agar (Potatoes 200.0; Dextrose 20.0; Agar 15.0), and Actinomycetes isolation agar (Sodium caseinate 2.0; L-Asparagine 0.10; Sodium propionate 4.0; Dipotassium phosphate 0.5; Magnesium sulphate 0.1; Ferrous sulphate 0.001; Agar 15.0) media of Himedia^®^ were used, respectively. The soil suspension of 10^-5^, 10^-4^, and 10^-3^ dilutions was poured on triplicate petri plates containing respective media for bacteria, fungi, and actinobacteria, respectively. Bacterial, fungal, and actinobacterial colonies appearing after 1, 3-4, and 6-7 days after incubation at 28 ± 2°C were counted, respectively.

The degree to which species or organisms in a sample are taxonomically or phylogenetically related to one another was determined using biodiversity indices. The microbial diversity index was calculated as described by Suchiang et al. (2020). The soil culturable microbial diversity was estimated using the following equation (Xia et al., 2022):


(vi) $$H=-\sum_{i=1}^{s}(Pi\times \log_{e}Pi)$$

Where, H= the Shannon diversity index for soil culturable microbial diversity, *Pi*= fraction of the entire population made up of microbial genus/group *i*, *s*=numbers of microbial genus/group encountered, ∑ =sum from species 1 to species *s*, log_e_ = the natural logarithm.

This index considers both the richness and evenness of microorganisms and provides equal weight to rare and common microbial genera/groups.

The soil culturable microbial dominance index (D) was estimated from the Simpson dominance index (D) using the following equation (Qianwen et al., 2022):


(vii) $$\text{D}=\sum_{i=1}^{s}P_{i}^{2}$$

The Simpson dominance index describes the most common species in the community. The higher value of the index suggests that few species are dominant in the community of species.

### 2.9 Nitrogen use efficiency indicators

Three approaches were used to quantify different N use efficiencies (NUEs), the N difference approach (Quan et al., 2021), the N balance approach (Quan et al., 2021), and agronomic efficiency (Fixen et al., 2015).

#### 2.9.1 NUE with nitrogen difference approach

NUE_diff_ is based on the difference between the N harvested in the fertilized treatment (NH_t_) and the N harvest in the non-fertilized control (NH_o_) treatment, then divided by N fertilizer (organic + inorganic) inputs (F_input_).


(viii) $${\text{NUE}}_{\text{diff}}\;=\;\;\frac{{\text{NH}}_{\text{t}}-{\text{NH}}_{\text{O}}}{{\text{F}}_{\text{inputs}}}$$

The major focus of NUE_diff_ assessment is on the fertilizer N recovery efficiency during a growing season with no consideration of legacy effects.

#### 2.9.2 Nitrogen balance approach

NUE_bal_ is based on the N harvested in any treatment (NH_t_) divided by all N inputs, including all fertilizer inputs (F_inputs_) and non-fertilizer inputs (NF_input_). We included the N inputs from fertilizer (organic + inorganic) for calculating F_inputs_, and N deposition (Mishra and Kulshrestha, 2022) for NF_inputs_.


(ix) $${\text{NUE}}_{\text{diff}}\;=\;\;\frac{{\text{NH}}_{\text{t}}}{{\text{F}}_{\text{inputs}}+{\text{NF}}_{\text{inputs}}}$$

The focus of NUE_bal_ assessment is on the use efficiency of all N inputs as well as the fraction of N inputs subject to loss. It is assumed that there is no change in the soil N status and it is in a quasi-steady state (Quan et al., 2021). In the current long-term experiment, the average season-to-season change in soil N stock was very small compared to the N inputs during the growing seasons.

#### 2.9.3 Agronomic efficiency

The agronomic efficiency (AE) was calculated based on the fertilizer N applied (AE_fN_) as well as based on total N applied (AE_tN_). These were calculated as


(x) $$AE_{tN}\;=\;\frac{\left(Y-Y_{O}\right)}{tN}$$

(xi) $$AE_{fN}\;=\;\frac{\left(Y-Y_{O}\right)}{fN}$$

Where Y is the crop yield in the treatment plots, Yo is the crop yield in the control (no fertilizer application), tN is the total amount of N (inorganic + organic) applied to a system, and fN is the amount of N applied through inorganic fertilizer.

### 2.10 Sustainable nitrogen management index

The sustainability of N management was calculated for different nutrient management strategies using the Sustainable Nitrogen Management Index (SNMI) proposed by Zhang et al. (2022b). The index takes into consideration the requirements for both food production and environmental conservation by combining the performance in N crop yield and N use efficiency (NUE). In the standard SNMI evaluation, the reference yield was set at 90 kg ha^-1^ (considering a globally averaged yield target for meeting food demand in 2050) and the reference NUE at 1.0 (considered to be the optimal NUE). Agricultural SNMI combined NUE and land use efficiency (crop yield), two essential efficiency metrics, into a single ranking score. The normalized NUE (NUE*) and normalized yield (NYield*) distances from a reference point in a two-dimensional graphic are calculated geometrically by SNMI.

Briefly, the *NUE* is defined as NUE divided by a reference NUE (*NUE_ref_* = 1), and NUE values > 1 are adjusted downward to avoid inflating the score due to the mining of soil N. The normalized crop yield is defined as the crop yield divided by a reference crop yield (*NYield_ref_* = 90 kg ha^-1^). Sustainable N management is reflected by SNMI values close to zero, as both yield and NUE approach their targets. The mathematical definition of SNMI is the Euclidean distance from an ideal point targeted for NUE and yield, and the equations for calculation are as follows:


(xii) $$\text{SNMI}=\sqrt{{\left(1-N\;Yield^{*}\right)}^{2}+{\left(1-NUE^{*}\right)}^{2}}$$

Where,


(xiii) $$NYield^{*}=\;\;\;NYield/NYield_{ref}(NYield\leq NYield_{ref})1(NYield>NYield_{ref})$$

(xiv) $$\begin{array}{l} NUE^{*}=\;\;\;NUE\;/\;NUE_{ref}(NUE\;\leq \;NUE_{ref})1(NUE_{ref}<NYield_{ref}\leq \;1)\\ 1-\left(NUE-1\right)(1<\;NUE\;\leq \;2)0(NUE\;>\;2) \end{array}$$

### 2.11 Crop yield

The rice and wheat crops were plot-wise harvested in October and March, respectively, and threshed to record grain and straw yields on a hectare basis.

### 2.12 Statistical analysis

SAS was used to statistically examine all of the data, including growth parameters. The separation of means was tested using Tukey’s honestly significant difference test in JMP 9.0 for all parameters (SAS Institute Inc., Cary, NC, USA). The graphing was done with the software Origin v.8.5 (Originlab Corporation, Northampton, USA). To detect correlations between the measured parameters, correlation analysis was used. All tests were carried out with a significance threshold of 0.05.

## 3 Results

**Figure 2:**
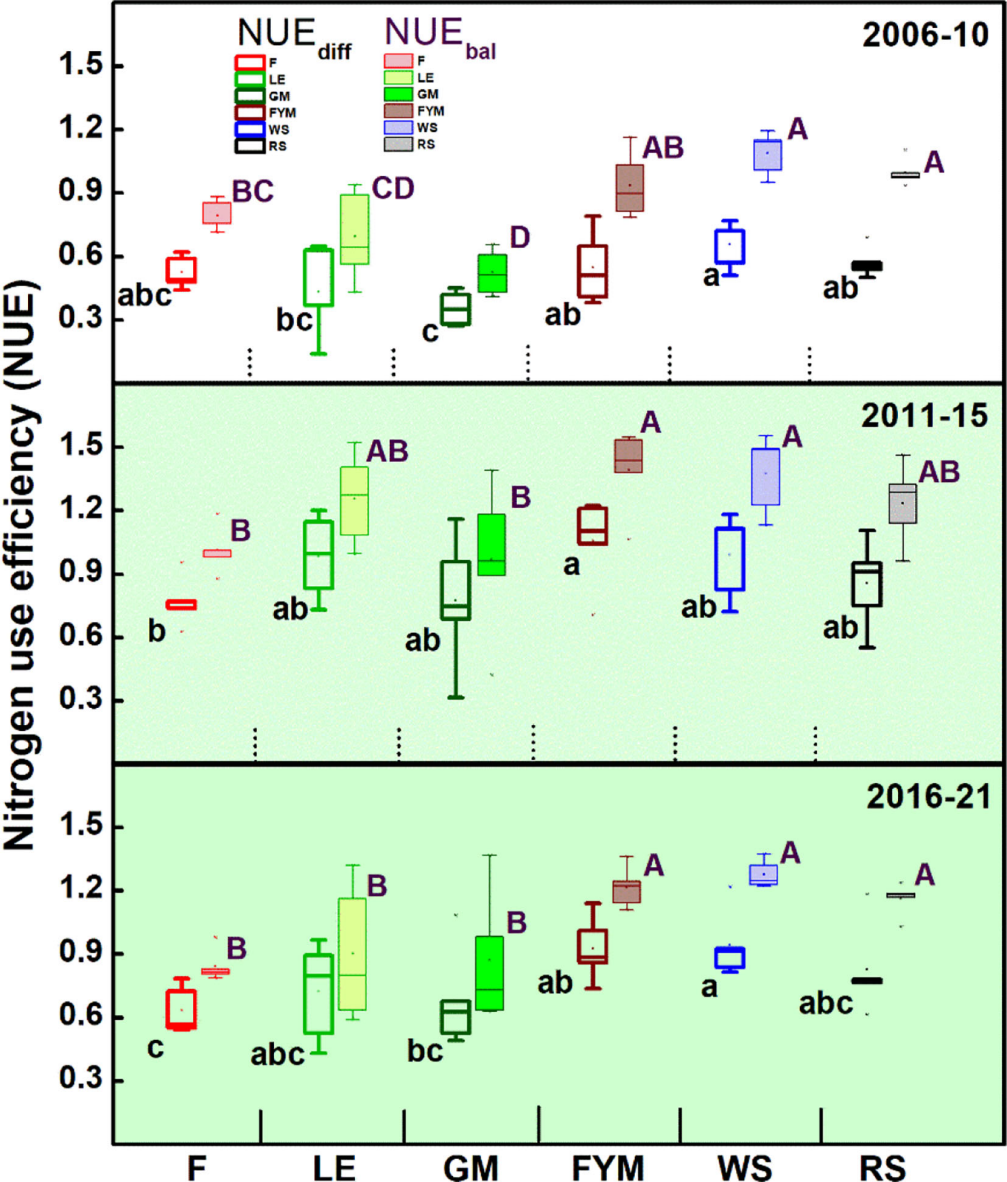
Trends in the nitrogen use efficiency (NUE) for rice crop during 15 years (2006-2021) of different nutrient management. Management: Error bars denote ± 1SD. Different letters (capital for NUE_bal_ and lower for NUE_diff_) indicate significant differences for NUE (P ≤ 0.05). O= no fertilizer, F= 100% inorganic fertilizer, LE= Legume (*Vigna radiata*) in rotation and its biomass incorporation + ~50% inorganic fertilizers, GM= Green manuring with *Sesbania esculeata* + ~50% inorganic fertilizers, FYM= farmyard manure incorporation + ~50% inorganic fertilizers, WS= 1/3 wheat stubble retention + ~50% inorganic fertilizers, RS= 1/3 rice stubble retention + ~50% inorganic fertilizers.

**Figure 3:**
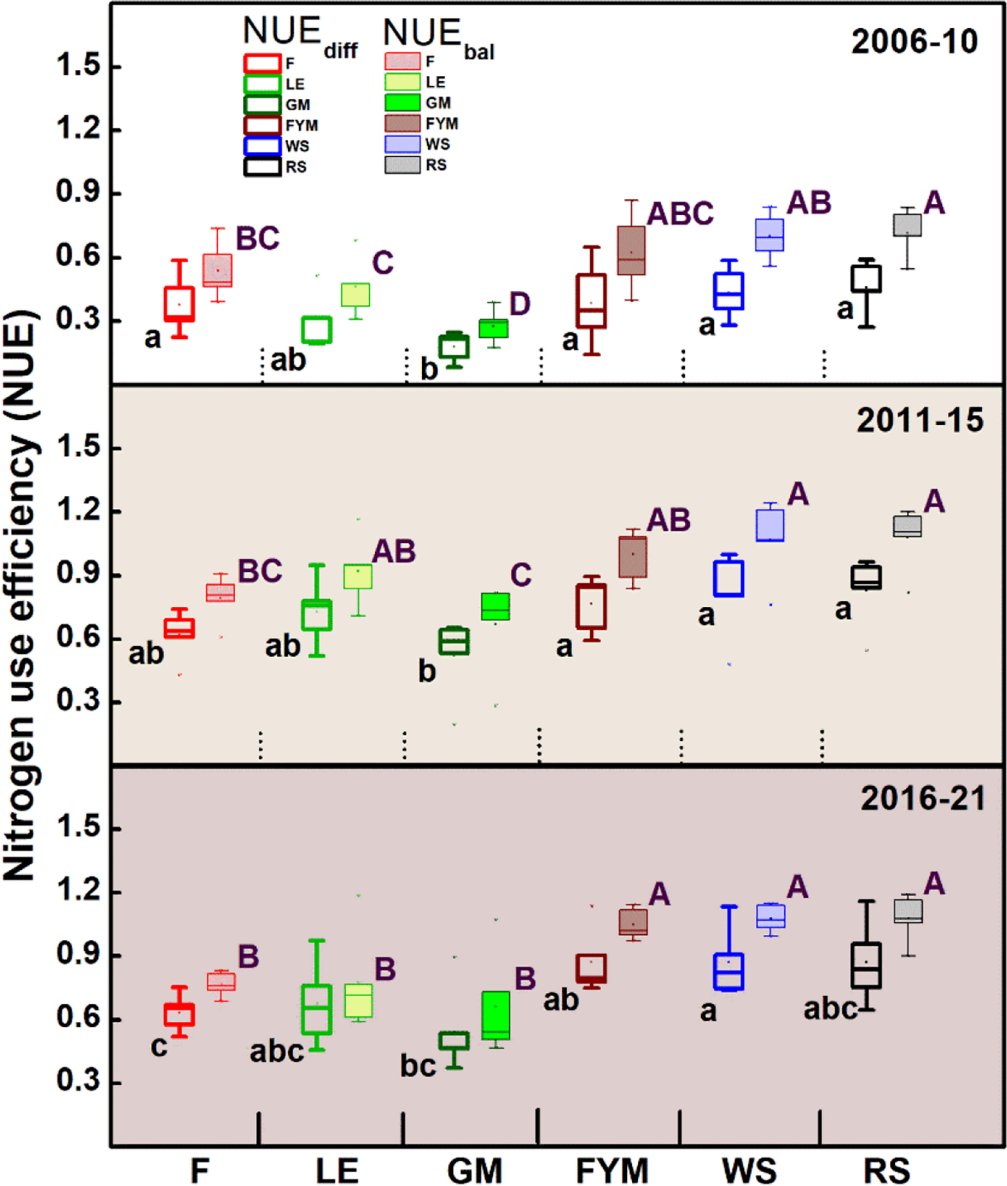
Trends in the nitrogen use efficiency (NUE) for wheat crop during 15 years (2006-2021) of different nutrient management. Error bars denote ± 1SD. Different letters (capital for NUE_bal_ and lower for NUE_diff_) indicate significant differences for NUE (P ≤ 0.05). Refer to **Figure 2** for the description of treatments.

**Figure 4:**
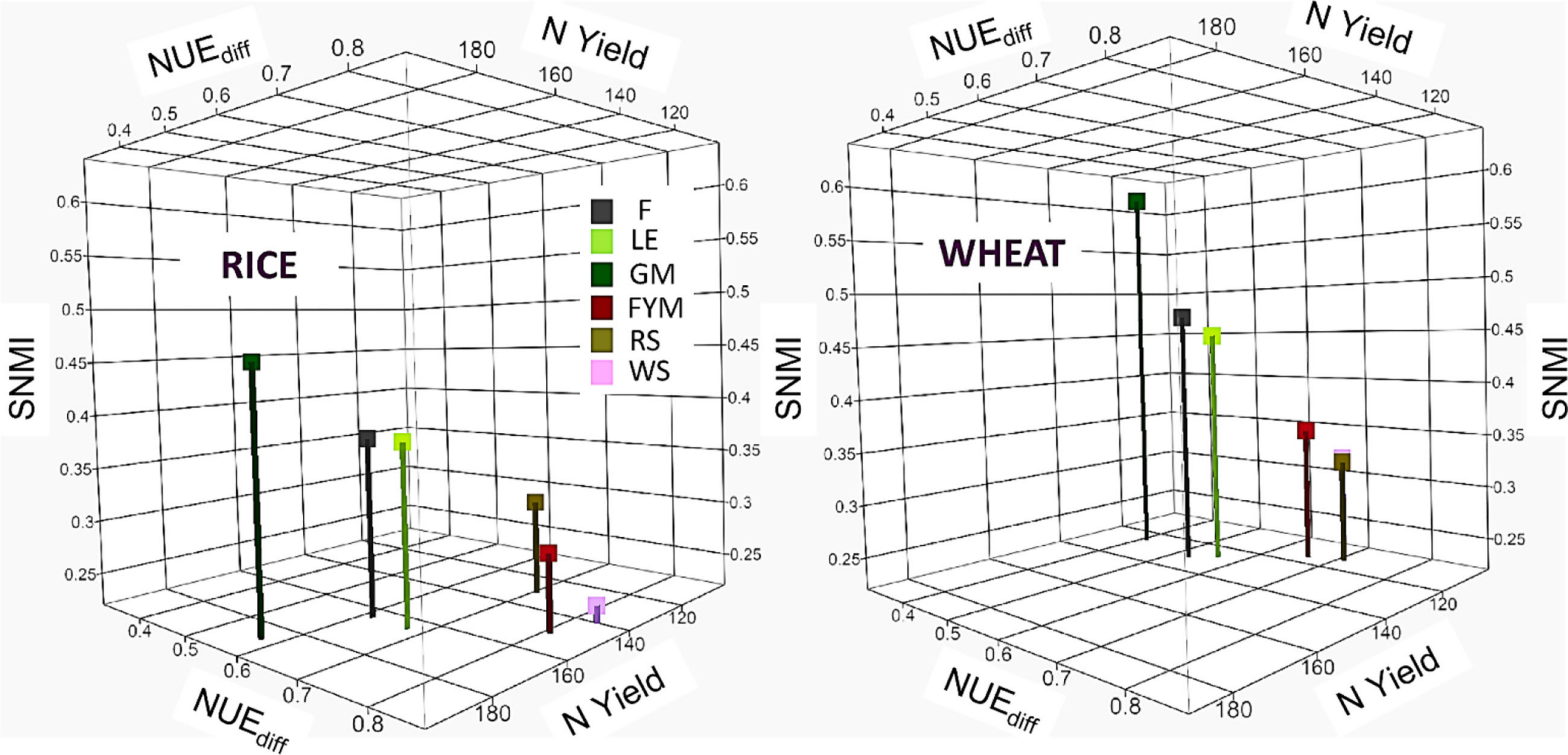
Fifteen-year (2006-2021) averages for the sustainable nitrogen management index (SNMI), nitrogen use efficiency (NUE_diff_), and the N yield (kg N ha^-1^ season^-1^) for rice and wheat crop under different nutrient management. Refer to **Figure 2** for the description of treatments.

Overall, around 190, 240,120, 120, and 110 kg ha^-1^ of N was added in the LE, GM, FYM, WS, and RS systems of integrated nutrient management (INM), respectively, compared to 180 kg ha^-1^ of N (all from chemical fertilizers) in F management (**Table 1**). At the same time, around 4.2, 7.0, 2.7, 2.9, and 3.3 Mg ha^-1^ of carbon (C) was also added each year through the organic inputs (*in situ* or *ex situ*). Broadly, for rice crop, the NUE_diff_ was highest in cereal crop residue and farmyard manure based INM (WS, RS, FYM) followed by legume based INM (LE, GM) and F (only chemical fertilizers), especially after the initial five-year period (**Figure 2**). NUE_bal_ which was slightly higher than NUE_diff_ followed similar trends. For wheat crop, which followed rice in the cropping sequence, slight variations were noted, with the highest NUE_diff_ in ES, RS, and FYM followed by LE, F, and lowest in GM (**Figure 3**). NUE_bal_ followed almost similar trends. An averaged sustainable nitrogen management index (SNMI) for years (2006-21) indicated the best performance for cereal crop residues and FYM based management (WS, RS, FYM) followed by LE, F, and GM, for both rice and wheat crop (**Figure 4**). For both crops, SNMI performances corresponded to the trends (averaged for 2006-21) in NUE_diff_.

**Table 1:** The nitrogen and carbon added to the soil through each management system (conventional and integrated nutrient management).

Treatment	Cropping Sequence	Nature of organic inputs	Nitrogen conc. of organic input	Nitrogen added to soil through organic inputs	Nitrogen added to soil through chemical fertilizers	Carbon added to soil through the system
			%	kg ha^-1^	kg ha^-1^	Mg ha^-1^
F	Rice-wheat	None	–	–	180	1.8 ± 0.17
LE	Rice-wheat-*Vigna radiata*	*Vigna* residue (*in situ* soil incorporated after pod harvest)	1.7 ± 0.03	88	100	4.2 ± 0.32
GM	Rice-wheat- *Sesbania aculeata*	*Sesbania* Biomass (*in situ* soil incorporated at 35 days after planting)	2.6 ± 0.50	142	100	7.0 ± 0.36
FYM	Rice-wheat	*Farmyard manure* (*ex situ* soil incorporated at rice transplanting)	0.6 ± 0.06	21	100	2.7 ± 0.19
RS	Rice-wheat	Rice stubble (*in situ* soil incorporated at wheat sowing)	0.3 ± 0.04	11	100	2.9 ± 0.42
WS	Rice-wheat	Wheat stubble (*in situ* soil incorporated at rice transplanting)	0.4 ± 0.07	17	100	2.3 ± 0.18

Treatment: F= 100% inorganic fertilizer, LE= Legume (Vigna radiata) in rotation and its biomass incorporation + ~50% inorganic fertilizers, GM= Green manuring with Sesbania esculeata + ~50% inorganic fertilizers, FYM= farmyard manure incorporation + ~50% inorganic fertilizers, WS= 1/3 wheat stubble retention + ~50% inorganic fertilizers, RS= 1/3 rice stubble retention + ~50% inorganic fertilizers.

**Figure 5:**
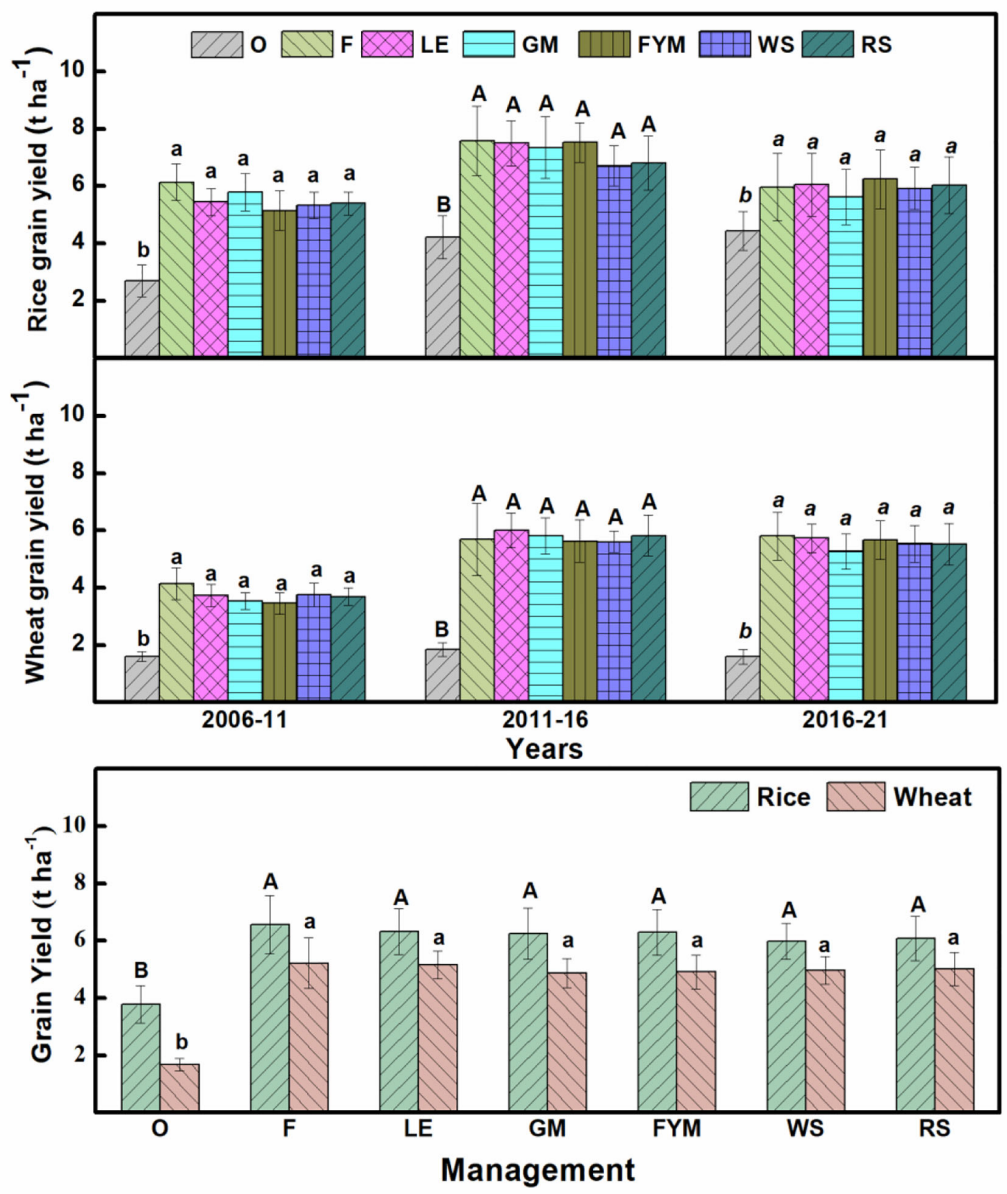
Trends in the grain yield (t ha^-1^) of rice and wheat crop during 15 years (2006-2021) of different nutrient management. Error bars denote ± 1SD. Different letters indicate significant differences (P ≤ 0.05). Refer to **Figure 2** for the description of treatments.

**Figure 6:**
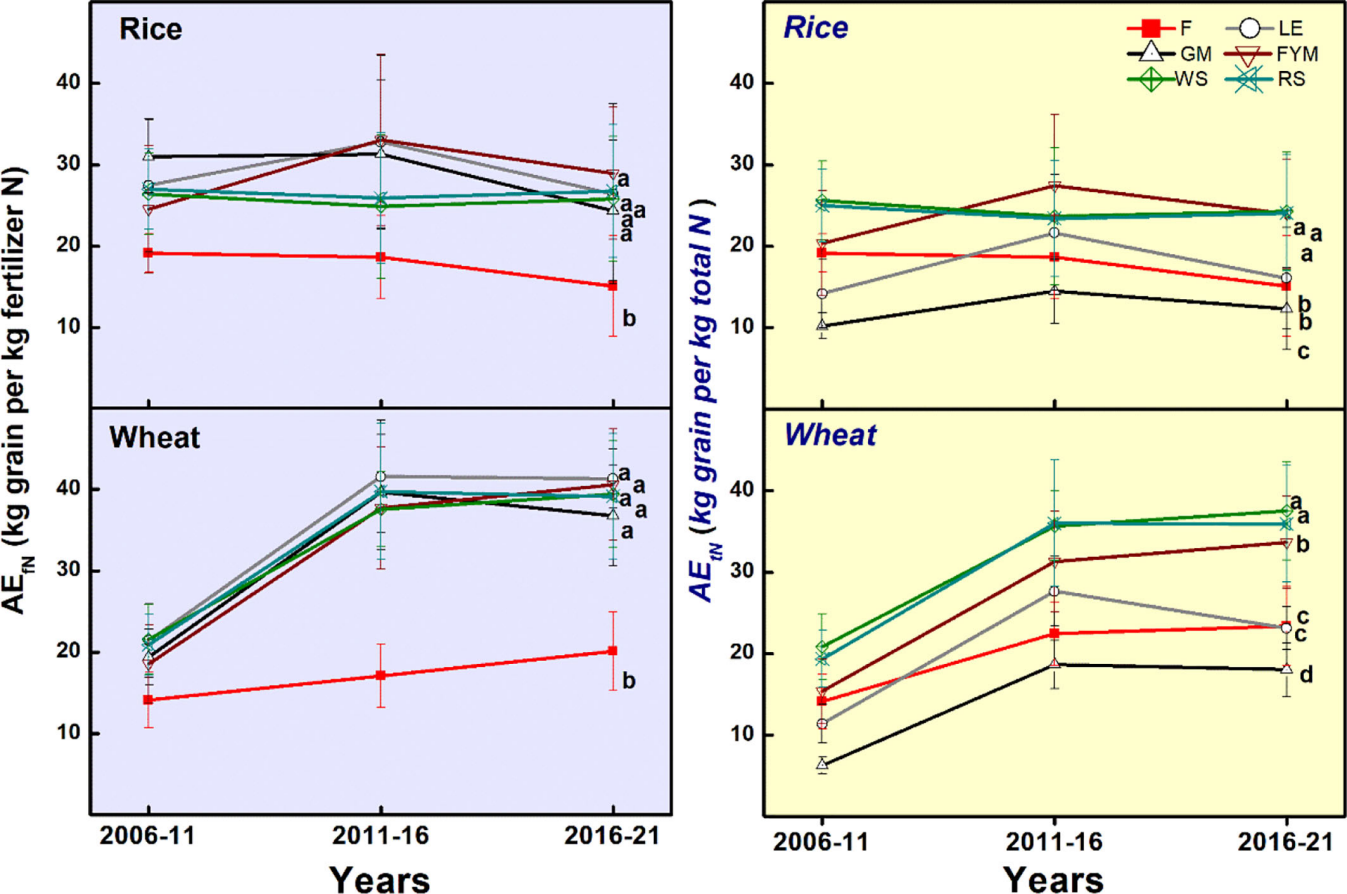
Trends in the agronomic efficiency (AE; AE_fN_ based on the chemical fertilizer N applied, and AE_tN_ based on the total N applied from all organic and inorganic sources) of rice and wheat crops during 15 years (2006-2021) of different nutrient management. Error bars denote ± 1SD. Different letters indicate significant differences (P ≤ 0.05). Refer to **Figure 2** for the description of treatments.

The five-year averages, as well as 15-year average for both rice and wheat crops, revealed the grain yield to be at par for all management (F, LE, GM, FYM, WS, RS) except where no fertilizer was applied (O) (**Figure 5**). For straw yield, there were significant (P ≤ 0.05) differences during different years for both rice and wheat crops (**Figure S1**). For rice crop, INM with the organic sources having high to medium N concentration (GM, LE, FYM) had significantly(P ≤ 0.05) higher straw yield compared to the cereal-residue-based INM with comparatively lower N concentration of organic components. For wheat, there were slight differences during the initial years but these were leveled from 5th year onwards. The agronomic efficiency (AE) showed no changes over the years for rice while there was an improvement in NUE for the wheat crop (15% initial average for all treatments to 30% after 15 years) (**Figure 6**). The management with integration of organics (INM) revealed significantly (P ≤ 0.05) better fertilizer AE_fN_, compared to F, for both rice and wheat. In the case of AE_tN_ (based on the N added from all inorganic and organic sources), the trends were similar to AE_fN_, over the 15 years of management. For both crops, the AE_tN_ was significantly (P ≤ 0.05) higher for the management where N concentration was low in the organic component of INM (WS, RS, and FYM). The AE_tN_ decreased as the N concentration increased. The AE_tN_ was in the order WS>RS> FYM> LE> F >GM.

**Figure 7:**
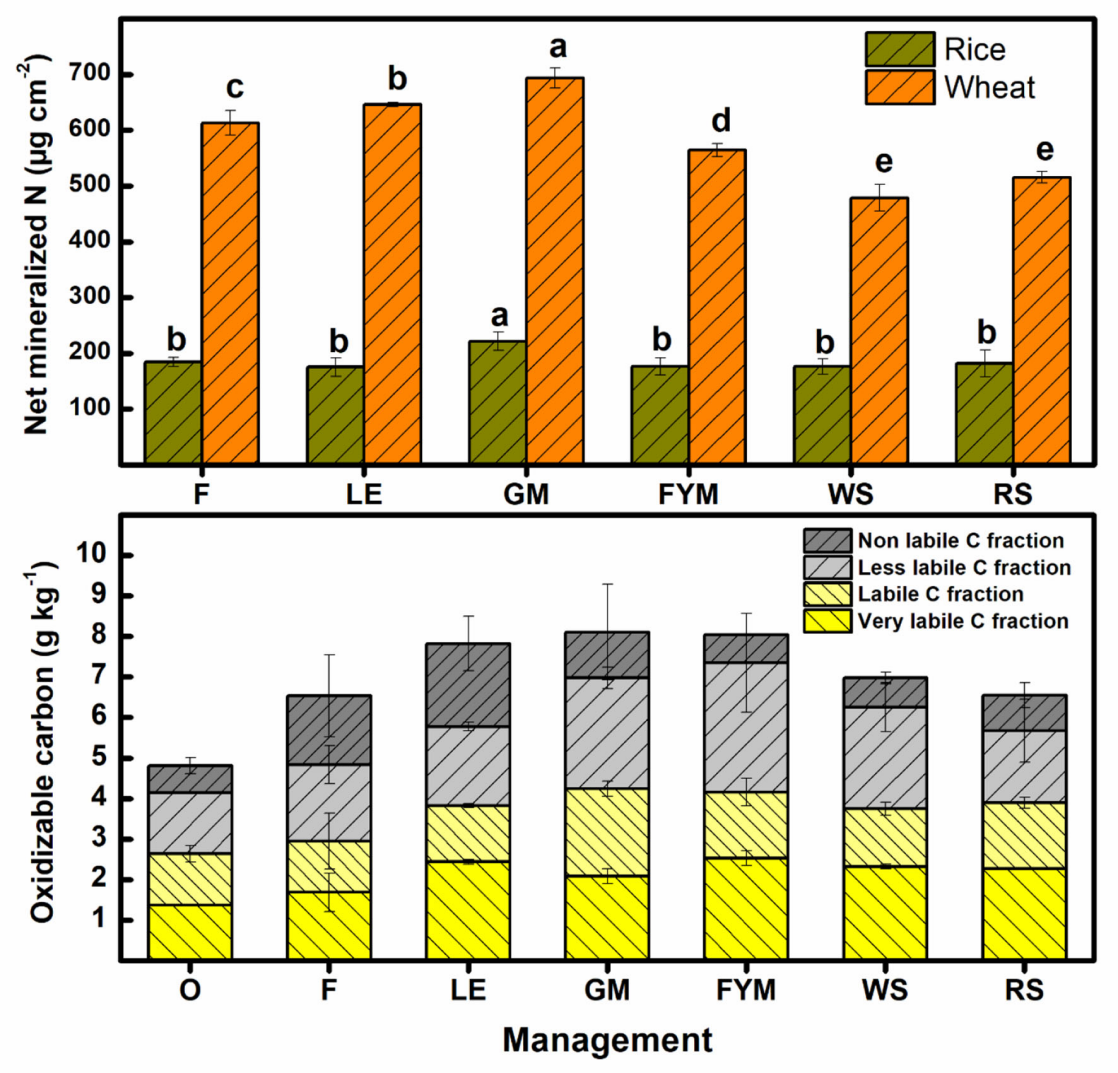
Effect of different nutrient management on net mineralized nitrogen during three full seasons of rice and wheat (2017-20), and the changes in soil carbon fractions. Error bars denote ± 1SD. Different letters indicate significant differences (P ≤ 0.05). Refer to **Figure 2** for the description of treatments.

During rice season, net mineralizable-N was highest in INM with green manuring (GM) than the rest of other management which were significantly (P ≤ 0.05) similar among themselves (**Figure 7**). Cereal-based INM management (WS, RS), and F had nearly 20 percent and 15 percent less mineralized N compared to GM during the full rice season. For the wheat crop, the net N mineralization during the full season was significantly (P ≤ 0.05) higher in integrated nutrient management with green manure (GM) followed by LE, F, and FYM. Least N- mineralization was noted in INM with cereal residues (WS, RS). The oxidizable carbon in soil depth up to 15 cm was significantly (P ≤ 0.05) higher in INM approaches with legumes (GM, LE) as well as farmyard manure (FYM), in comparison with cereal residue-based INM strategies (WS, RS), and F (**Figure 7**). Among different fractions of carbon, non-labile C (NLc) fraction was maximum in INM with LE management followed by others. Whereas, less labile C (LLc) fraction sequestered maximum in INM with FYM followed by GM, LE, WS, RS management, and least was accumulated in O. The labile C fraction was maximum in nutrient management with green manure (GM) followed by the rest of other management. Labile C fraction was in the order of GM>FYM>RS>WS>F>O. Overall, INM with GM had a maximum Lc fraction, while NLc fraction was maximum in LE management. Both LLc and VLc fractions had maximum buildup in FYM management.

**Figure 8:**
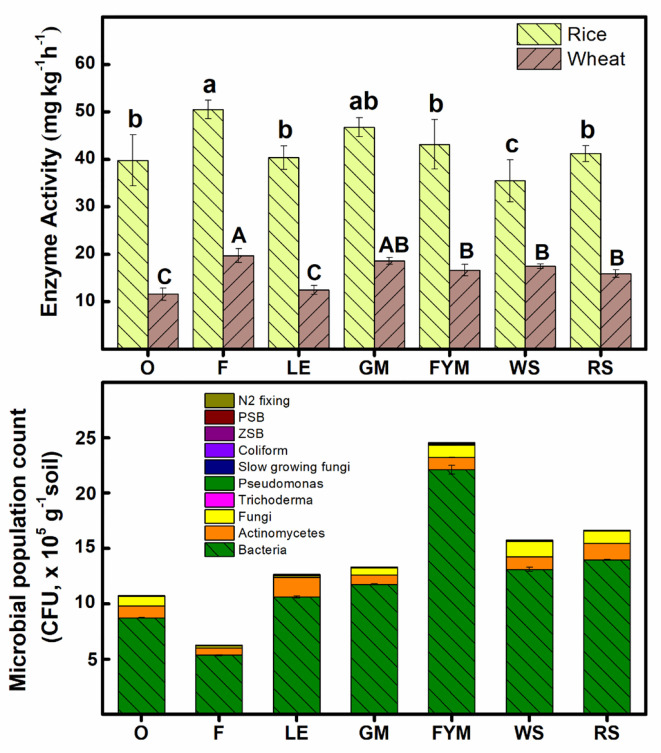
Effect of different nutrient management practices on the aggregated soil enzymatic activity, and soil microbial population. Error bars denote ± 1SD. Different letters indicate significant differences (P ≤ 0.05). Refer to **Figure 2** for the description of treatments.

For both rice and wheat crops, the aggregated enzymatic activity (mg kg^-1^h^-1^) was significantly (P ≤ 0.05) higher in F and GM management, compared to other management strategies (**Figure 8**). In the case of the rice crop, there were no significant (P ≤ 0.05) differences in enzymatic activity under FYM, LE, and RS management. Integrated nutrient management with retention and incorporation of wheat stubble (WS) had the lowest enzymatic activity. In the wheat crop, the enzymatic activity was similar under FYM, WS, and RS treatment. The least enzymatic activity was noted where no fertilizer was applied during the wheat crop. Among different INM strategies, FYM management had a maximum total microbial population count, compared to the rest of the management while it was minimum in F (**Figure 8**). The microbial population was in the order of FYM>RS>WS>GM>LE>O>F. However, cereal-based INM management (RS, WS) had a higher microbial population count, compared to legume-based (GM, LE) INM management. The population of bacteria, PSB, ZSB, and slow-growing fungi was found to be highest in FYM management. Fungi and N-fixing microbes were maximum in nutrient management with wheat stubbles (WS). However, the population of actinomycetes, coliform, Pseudomonas, and Trichoderma were maximum in legume-based (LE) INM management. The soil culturable microbial diversity index was significantly(P ≤ 0.05) higher in WS ad RS management (as well as O management) compared to other treatments. FYM management had the lowest diversity index (**Figure S2**). However, the soil culturable microbial dominance index was highest in FYM management followed by GM and F management. The least diversity was noted under F management.

**Figure 9:**
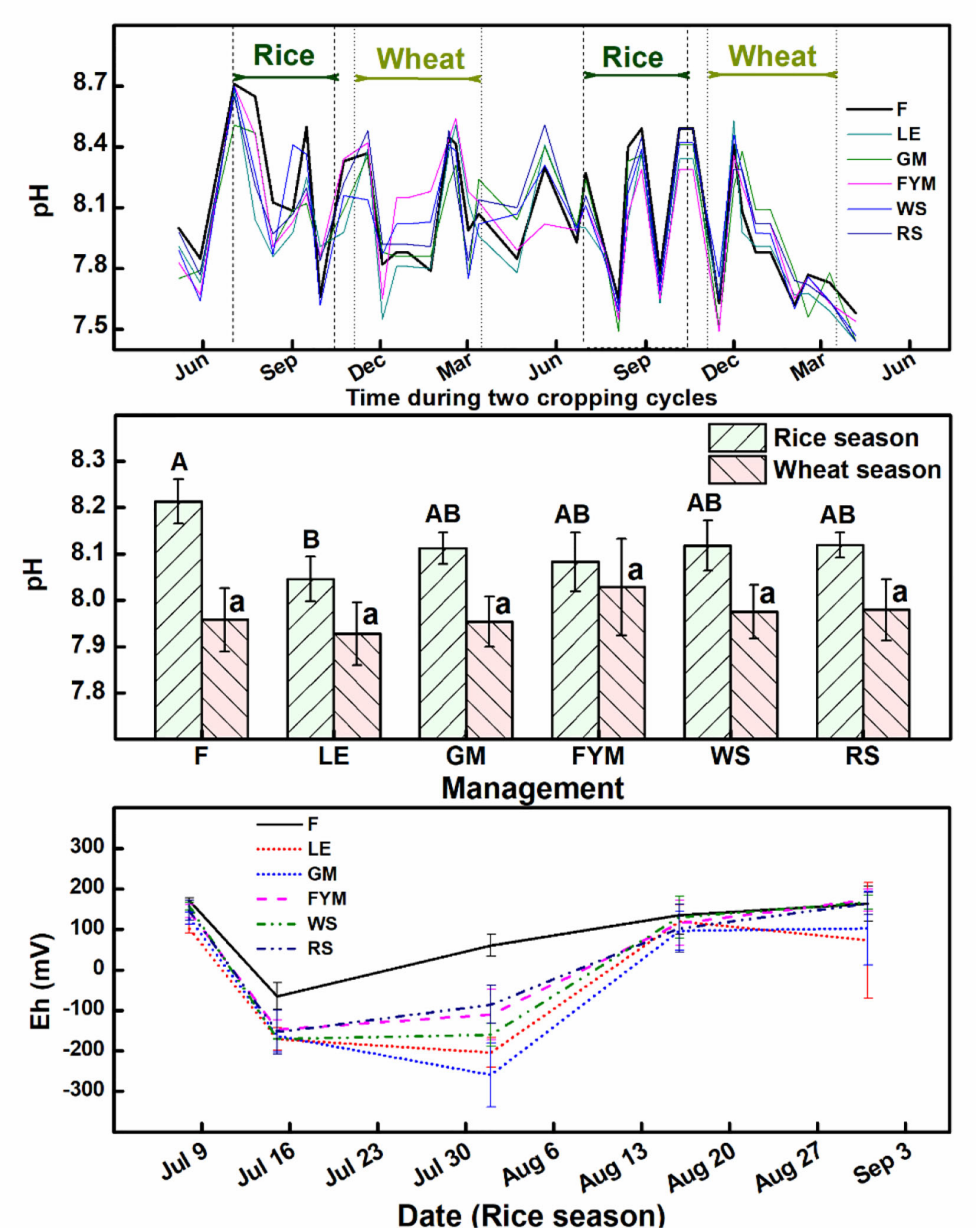
Effect of different nutrient management practices on soil pH and redox potential (Eh) during the rice-wheat seasons. Error bars denote ± 1SD. Different letters indicate significant differences (P ≤ 0.05). Refer to **Figure 2** for the description of treatments.

**Figure 10:**
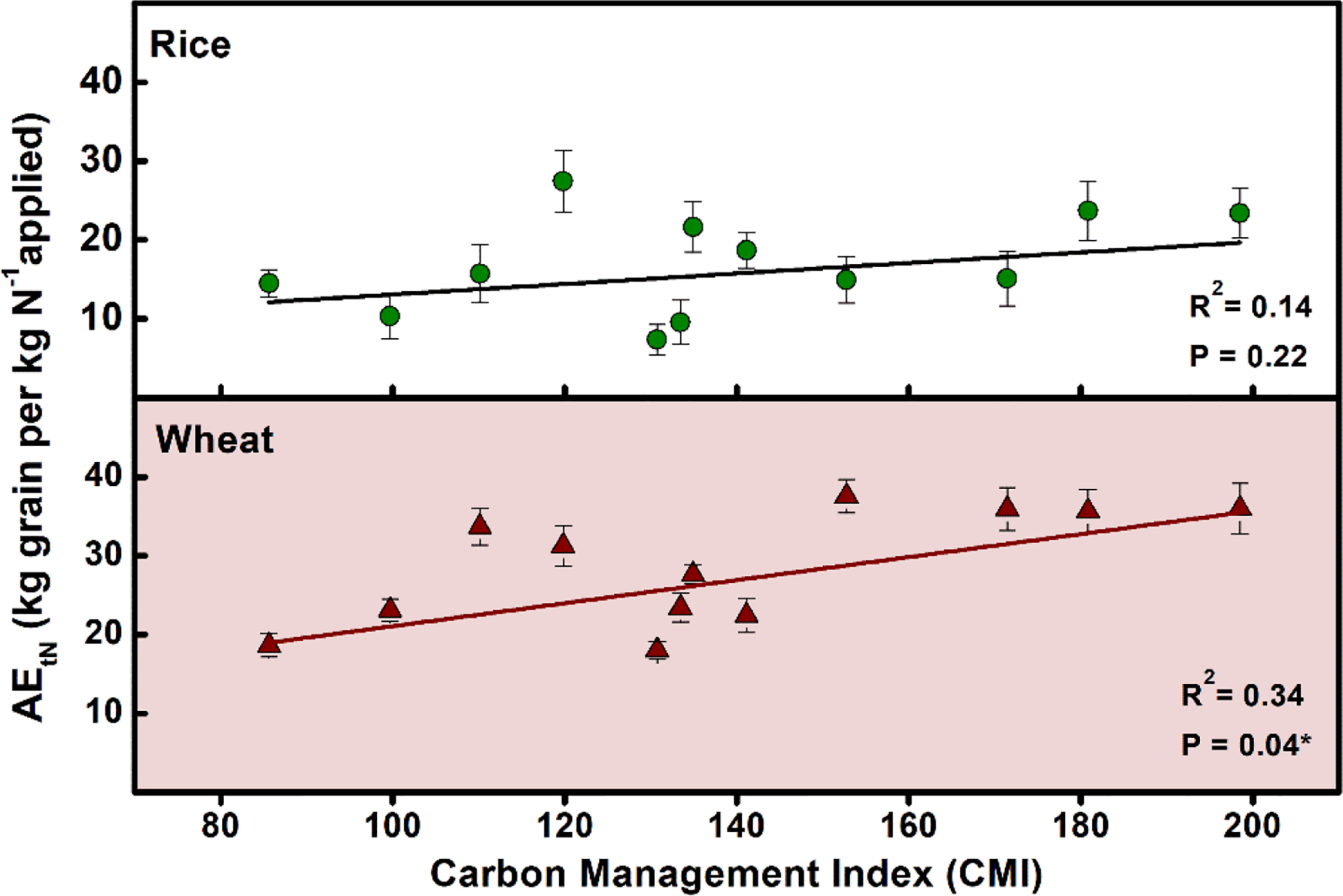
Relations of soil organic carbon (OC) and carbon management index (CMI) with nitrogen use efficiency under rice-wheat systems. Error bars denote ± 1SD. Refer to **Figure 2** for a description of treatments.

There were significant (P ≤ 0.05) changes in the electrochemistry of the soil, particularly during the rice season, which influences the N mineralization and plant availability. There was a noticeable drop in the pH of the soils in the INM treatments compared to F (**Figure 9**). There was also a significant (P ≤ 0.05) drop in the redox potential (Eh) after the flooding of soils and transplanting of rice, particularly for INM treatments. This drop was more for the legume-based management than the cereal residue-based management. The drop was more significant (P ≤ 0.05) for the first month but persisted until the ponding of water in the rice crop continued. Carbon management index (CMI), an indicator of more active carbon fractions, was positively and directly related to AEtN; AEtN increased as CMI increased (**Figure 10**). In the case of the wheat crop, the relationship was significant (P=0.04*), however in the case of the rice crop, it was not significant (P=0.22). A correlation matrix was generated between several soil parameters, yield and the nitrogen use efficiency (NUE) under tested managements (**Table 2**). In rice crop, sustainable nutrient management index (SNMI) (r = -0.95***, P<0.001), and enzyme activity (r = -0.766**, P<0.01) were negatively and highly significantly correlated with NUE. The relationship between NUE and C-labile factions (r = 0.703*, P<0.05), bacterial community (r = 0.687*, P<0.05), fungal community (r = 0.748*, P<0.05), slow growing fungi (r = 0.792**, P<0.01), and N_2_ fixing bacterial community (r = 0.733*, P<0.05) was also found positive and significant. In case of wheat crop, highly significantly relationship was revealed between sustainable nutrient management index (SNMI) (r = -0.905***, P<0.001; for SNMI lower values indicate better N management) and NUE. Whereas, N yield had positive and significant correlation (r = 0.836**, P<0.01) with C non-labile fraction, but a negative correlation with fungi (r = -0.957*, P<0.05) and slow growing fungi (r = -0.835**, P<0.01). For both rice and wheat crops, grain yield was positively and highly significantly correlated with straw yield (r = 0.836***, P<0.0011; r = 0.957***, P<0.001) and but less significantly correlated with C very labile fraction (r = 0.686*, P<0.05; r = 0.726*, P<0.05).

**Table 2:** Correlation matrix of various soil chemical and biological parameters with grain yield, and nitrogen use efficiency parameters (N use efficiency-NUE, sustainable N management index-SNMI, N-yield) recorded under different tested nutrient management in rice wheat system.

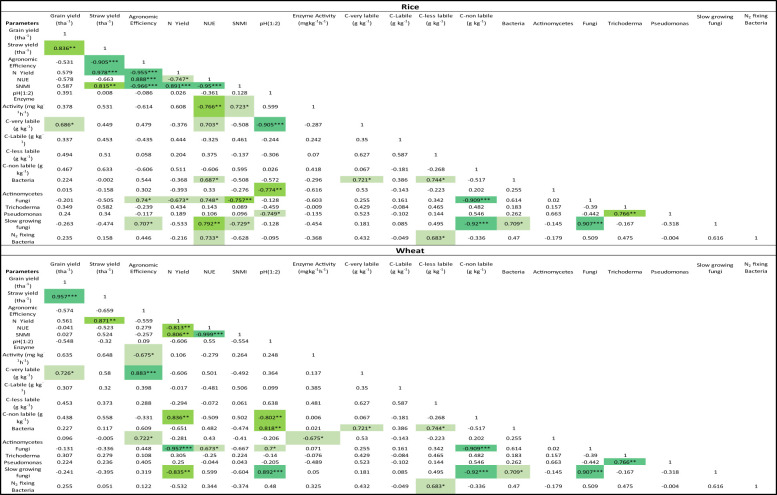

(*P<0.05, ** P<0.01, *** P<0.001).

## 4 Discussion

In general, the nitrogen use efficiency (NUE) revealed that rice had 30-60% higher NUE_diff_ and 20-40% higher NUE_bal_, compared to wheat crop. These differences decreased over time, especially for INM-based management. In general, higher NUE was obtained in the integrated managements along with substantial gain in sustainable N management index (SNMI). There were substantial improvement in N mineralization, soil carbon, and enzymatic activity in legume and manure based management. A significant decrease in soil pH and redoc potential was noticed in rice soils in all management with integration of organics. While there was no noticeable yield gain in the INM strategies (LE, GM, FYM, WS, RS) compared to F (only chemical fertilizers), the nitrogen use efficiencies were significantly (P ≤ 0.05) higher in the former indicating that ~50% cut (100 kg ha^-1^ instead of 180 kg ha^-1^) in the chemical fertilizer use was affordable without any loss in yield. The INM strategies where low- N organic matter (cereal crop residue, FYM; ~ 0.5% N) was added, along with ~ 50% of recommended inorganic fertilizers gave the best NUE followed by those with medium N organic matter (grain legume crop; ~ 1.5% N), and it was lowest in the high N organic matter (green manure legume; ~2.5% N). Though N use efficiency (NUE) is a combination of several plant and agronomy related determinants, the processes in soil have a significant role to play *via* influences on mineralization- loss- plant uptake synchrony (Bhardwaj et al., 2020a). While the uptake of N by plants is driven by the transpiration-related mechanisms (Chen et al., 2020), the rate of N mineralization, plant uptake, and loss, the three simultaneously occurring processes play a major role in determining the NUE of a crop production system (Bhardwaj et al., 2021). Globally, the NUE has hardly exceeded 40% NUE (Omara et al., 2019; Chivenge et al., 2021). Organic sources of nutrients have a slow release which often mismatches with plant demand, especially at critical stages when the need is high while the use of sole chemical fertilizers provides quick mineralization (to available forms of ${\text{NH}}_{4}^{+}$ and ${\text{NO}}_{3}^{-}$ ) as well as quick loss *via* leaching ( ${\text{NO}}_{3}^{-}$ ) and volatilization (NH_3_) or emission (N_2_O) (Ruan et al., 2016; Bhardwaj et al., 2021). Poor synchrony between demand and supply as well as slow mineralization has been often analyzed to be the cause of low NUE, particularly in the organic system (Alaru et al., 2014; Musyoka et al., 2017). Integrated nutrient management (INM) systems have been noted to provide better NUE (Dwivedi et al., 2016) yet mechanisms are not very clear, except for controlling water-management-based leaching losses.

Integration of organics in nutrient management has advantages for both organic and inorganic fertilization systems, and it has been shown to have a consistent release of N (Bhardwaj et al., 2020a). Rice and wheat crop have contrasting management, and the N losses are also *via* different channels. Ammonia (NH_3_) Volatilization is a dominant N loss channel in flooded rice while nitrate ( ${\text{NO}}_{3}^{-}$ ) leaching and loss is a significant loss in wheat (Bhardwaj et al., 2020b). Nitrogen use efficiency in the rice-wheat system as a whole, therefore, has much to do with the mineralization mechanisms under anaerobic (rice) and aerobic conditions (wheat). Redox potential and pH have been noted to be major drivers of fertility for submerged rice (Sahrawat, 2015), and improvement in these can provide an advantage for NUE. The total N mineralization in the rice season had distinct similarities for all systems except GM but the N mineralization varied significantly (P ≤ 0.05) among INM in the wheat season (**Figure 7**). In INM, organic components with high N concentrations had higher total net mineralization than those with low N concentrations. During the wheat season, net N mineralization was higher while NUE was lower compared to rice. Rice is a three-month crop (after transplanting) while wheat is a five-month crop. The aerobic conditions during wheat crop had ~1.5 times higher daily net N mineralization rate as well as ~2-3 times more full-season N mineralization than rice crop, indicating that the losses in wheat crop could be substantial despite dry-upland conditions during most of the growing period. Intermittent rain or irrigation waters may carry a significant amount of nitrate ( ${\text{NO}}_{3}^{-}$ ) which is a highly mobile form of N as it does not bind to soil (Subbarao and Searchinger, 2021). The nitrate leaching and release of N_2_O due to the breakdown of ${\text{NO}}_{3}^{-}$ by microorganisms add to greater losses and low NUE during the wheat season. Therefore, if the NUE of the rice-wheat system needs to be improved then improvements in N mineralization and release characteristics and curtaining losses during the wheat season could be an important focus point. In that case, the INM strategies wherein organic materials with more recalcitrant carbon and wider C: N ratios are used would provide better NUE, provided that enough N is mineralized to meet plant demand at critical stages. This was also noted in the current study wherein significant (P ≤ 0.05) improvement in NUE was noted for cereal crop residue based (WS, RS) and FYM based INM strategies (**Figure 3**). The advantage of integrated management could be that the immediate N requirement at critical stages can be met by the inorganic fertilizer applied at reduced rates. Otherwise, a wider C: N ratio may hinder N availability to plants if mineralized N is not available in soil or externally supplied at critical stages. All systems tested in the study had N application (whether 100% or ~50%) in three equal splits at 0, 21, and 42 days after transplanting (rice)/sowing (wheat) of the crop.

Since the N mineralization-losses-plant uptake nexus play the key in determining the NUE of the rice-wheat system, the changes in soil biological characteristics, particularly those related to soil carbon and decomposition would play an immense role. Whether, it is electrochemical changes, soil carbon, enzymatic activity, or microbial population characteristics, efficient use of N would be affected by the characteristic influence of these changes on mineralization rate, adsorption and retention, losses *via* leaching and volatilization, and uptake by the plants *via* an effect on root proliferation. Soil enzymes play a vital role in the biochemistry of organic matter decomposition and nutrient mineralization by breaking down complex organic compounds. The quality of organic materials/residues, enzymes, and other biological forms in soil work together to make plant nutrients available (Srinivasa Rao et al., 2017). Thus, the combination of N and C added through an INM practice would also produce diverse responses, as noted in the current study. The green manuring based management (GM), with high total-N and less-recalcitrant carbon inputs, had higher enzymatic activity than the WS, RS, and FYM with comparatively lesser N concentration and highly recalcitrant C. The latter (WS, RS, and FYM) gave higher NUE than the former.

Notable trends in redox potential (Eh) and pH were also noted with the addition of organic materials, particularly during the rice season. More than two months of full flooding during the rice season can lead to a redox system wherein the losses of N can be large *via* ammonia loss (ammonification), ${\text{NO}}_{3}^{-}-\text{N}$ loss (nitrification), and N_2_O losses (denitrification). These electrochemical changes have important relations to nitrogen availability and uptake by rice plant (Bhardwaj et al., 2020b). One major effect of the reduction in Eh is that most of N mineralization is up to the ${\text{NH}}_{4}^{+}-\text{N}$ formation, and ${\text{NO}}_{3}^{-}-\text{N}$ is unstable under reduced conditions, though the conditions may not be perfectly reduced in the open paddy fields (Bhardwaj et al., 2020a). The availability of N in ammoniacal forms for a longer time ensures better plant uptake and N use efficiency (Subbarao and Searchinger, 2021). Studies on the effect of redox potential by Minick et al. (2016) indicated that an increase in redox increases dissimilatory-nitrate-reduction-to-ammonia (DNRA) and decreases N_2_O emission, thus reducing N losses. These pieces of evidence support the results of the current study. The changes in electrochemistry in rice soils, decrease in redox potential and neutralization of alkalinity, would help in enhancing nutrient availability and use efficiency (Sahrawat, 2015; Bhardwaj et al., 2020b).

Another major influence on the NUE could have been due to the changes in soil carbon with the integration of organics in the tested INM strategies. Highly significant (P ≤ 0.05) trends were noticed in the relation between carbon management index (CMI) and agronomic efficiency (AE_tN_, AE_fN_), particularly for the wheat crop which is grown under relatively drier conditions than rice. Labile (active pool) organic C fractions in soil seem to contribute to improved NUE significantly, particularly for wheat crop, our results indicate. The role of active/labile soil C fractions in influencing the profitability of fertilizer use has been realized more recently (Chamberlin et al., 2021). Active fractions of soil C are more strongly related to soil functions, such as nutrient cycling (Chamberlin et al., 2021), soil aggregation, and soil quality (Bembi et al., 2015). Bhardwaj et al. (2019) also noted increased resilience of crops in systems with more soil C sequestration. The carbon management index (CMI) is an indicator of carbon storage in form of active/labile fractions. Less labile (passive pool) soil C fractions which are although important for C sequestration in the soil to achieve climate change mitigation goals seem to play a lesser role in NUE enhancement. An increase in CMI has also been advocated to be an indicator for C rehabilitation of soils (Bembi et al., 2015), yet it appears to be a good indicator of improved NUE, at least for rice-wheat systems. Besides N mineralization, C mineralization has been related strongly to plant growth and yield (Culman et al., 2013). Many previous studies have highlighted the connection between N mineralization and C mineralization, and the significance of this relationship for improved crop performance (Vahdat et al., 2010; Sherrod et al., 2012; Culman et al., 2013).

## 5 Conclusion

Nitrogen use efficiency (NUE) of a cropping system is a response to its agronomic management mediated by soil conditions and nitrogen transformations favoring plant growth. Since rice and wheat have different management, with anaerobic-water flooded-puddled soils in the rice season and aerobic-dry tilled soils in the wheat season, enhancing efficiency in both types of conditions can improve the system’s overall NUE. Integration of organics along with the reduced rates of chemical fertilizers improved biological health, produced favorable electrochemical responses, increased soil carbon, and resulted in improved N mineralization dynamics. The electrochemical changes, such as the decrease in redox potential and pH during the rice season, boosted by the integration of organics, could help rice plants take up N more efficiently. For wheat crop, changes in labile soil carbon, as indicated by the carbon management index, correlated best with the improved NUE. The labile soil organic carbon was the most significant factor for improvement in NUE for rice and wheat crop, though the effects were more pronounced and significant in wheat. With better NUE in rice compared to wheat, the focus should be on the wheat crop to improve efficiency perhaps by prolonging retention of N as ${\text{NH}}_{4}^{+}-\text{N}$ in soil, and by achieving yield gains. Increasing the intensity of the redox process with the integration of organics can help immensely in improving NUE in transplanted-flooded rice. Green manuring with *Sesbania aculeata*, planting with grain legumes (*Vigna radiata*) and recycling their residue, and retaining and incorporating cereal crop residue were all found to significantly increase NUE in the rice-wheat system. Improvements in the overall biological health and changes in the electrochemistry of soil *via* these INM strategies can offset the need for almost 50% of chemical fertilizer N currently being used.

## Data availability statement

The original contributions presented in the study are included in the article/**Supplementary Material**. Further inquiries can be directed to the corresponding author.

## Author contributions

All authors contributed to the study’s conception and design. Data analysis, preparing visualizations, and writing the original draft were done by Ajay Kumar Bhardwaj, Kapil Malik, and Sukirtee Chejara. Deepika Rajwar, Bhaskar Narjary, and Priyanka Chandra helped in various analyses and data processing. All authors reviewed and edited the draft. All authors approved the final manuscript. Many scientists and technical personnel recorded agronomic data in this long-term project running since 2005 at CSSRI, Karnal, India. The authors acknowledge their contributions to collecting agronomic data and maintaining these records.
