# Supplementary material for: Integration of organics in nutrient management for rice-wheat system improves nitrogen use efficiency via favorable soil biological and electrochemical responses
Source: Front Plant Sci. 2023 Jan 5;13:1075011. doi: 10.3389/fpls.2022.1075011 (PMC9849818; doi:10.3389/fpls.2022.1075011)
Supplement: Supplementary file 1 [file DataSheet_1.docx]

**Integration of organics in nutrient management for rice-wheat system improves nitrogen use efficiency via favorable soil biological and electrochemical responses**

Ajay Kumar Bhardwaj*, Kapil Malik, Sukirtee Chejara, Deepika Rajwar, Bhaskar Narjary, Priyanka Chandra, Awtar Singh

Central Soil Salinity Research Institute, Karnal-132001, Haryana, India

**Corresponding author:**

Ajay Kumar Bhardwaj, Principal Scientist, Central Soil Salinity Research Institute, Kachhwa Road, Karnal 132001, Haryana, India. Tel: (+91) 184-2209359, Fax: (+91) 184-2290480, e-mail: [ak.bhardwaj@icar.gov.in](mailto:ak.bhardwaj@icar.gov.in)

**Contacts of co-authors:**

Kapil Malik, Senior Research Fellow, [kapilmalik017@hau.ac.in](mailto:kapilmalik017@hau.ac.in)

Sukirtee Chejara, Senior Research Fellow, [spsukirtee35@gmail.com](mailto:spsukirtee35@gmail.com)

Deepika Rajwar, Senior Research Fellow, deepika.rajwar@gmail.com

Bhaskar Narjary, Scientist, [bhaskar.Narjary@icar.gov.in](mailto:bhaskar.Narjary@icar.gov.in)

Priyanka Chandra, Scientist, priyanka.[chandra921@gmail.com](mailto:chandra921@gmail.com)

**

**

**Fig. S1** Five years trend and combined effect on straw yields (t ha^-1^) of rice and wheat crop for 15 years (2006-2021) of nutrient management in rice wheat system. Management: O= no fertilizer, F= 100 % inorganic fertilizer, LE= Legume (*vigna radiata*) in cropping sequence and its biomass incorporation + ~50% inorganic fertilizers, GM= Green manuring with *Sesbania esculeata* + ~50% inorganic fertilizers, FYM= farmyard manure incorporation + ~50% inorganic fertilizers, WS= 1/3 wheat stubble retention + ~50% inorganic fertilizers, RS= 1/3 rice stubble retention + ~50% inorganic fertilizers. Error bars denote ± 1SD. Different letters indicate significant differences (P≤0.05).

**
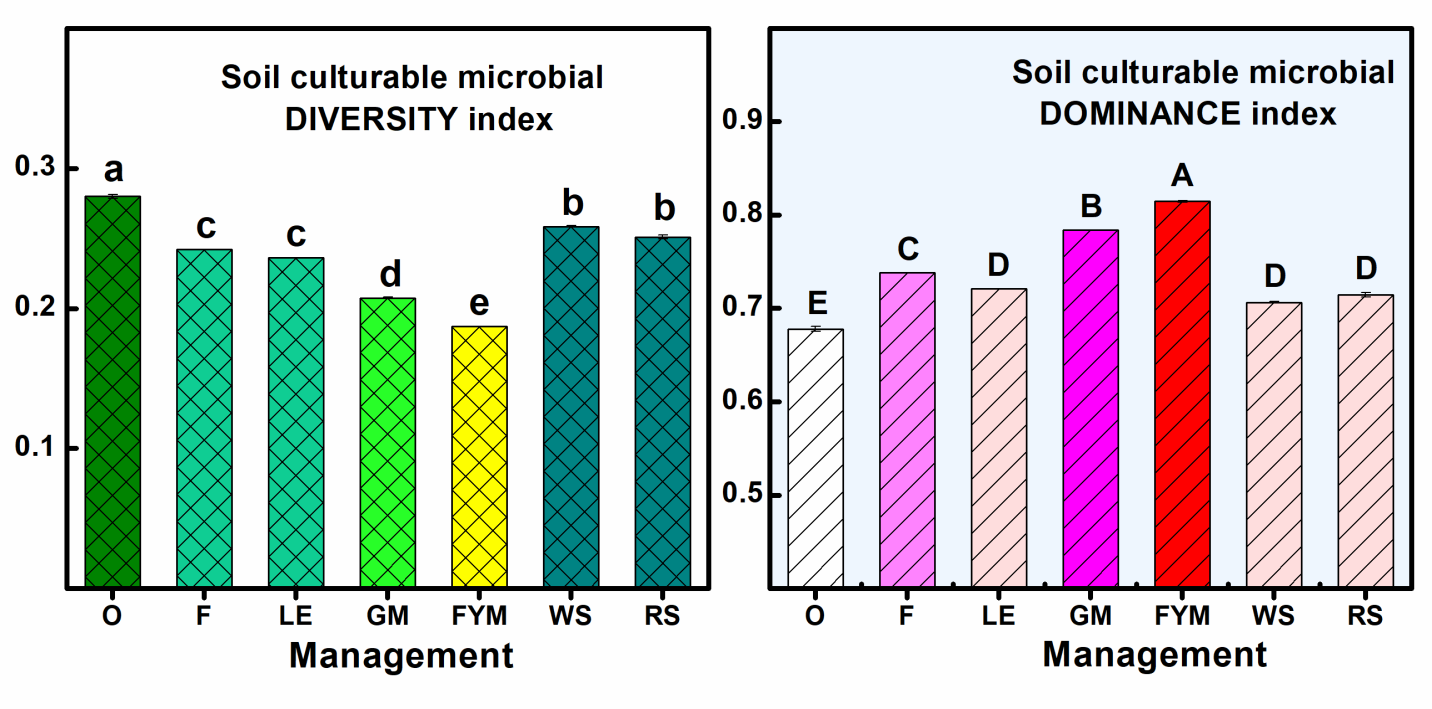
**

**Fig. S2** Effect of different nutrient management practices on soil microbial population (CFU, × 10^5^ g^-1^ soil) under rice-wheat system. Effect of different nutrient management practices on soil culturable microbial diversity and dominance index in rice-wheat system. Different letters indicate significant differences (P≤0.05). Error bars denote ± 1SE. Refer to Fig. S1 for a description of treatments.
